# Editorial: Cognitive schemas in primary headache disorders

**DOI:** 10.3389/fneur.2023.1240559

**Published:** 2023-07-12

**Authors:** Aynur Özge, Massimiliano Valeriani, Derya Uluduz, Vincenzo Guidetti

**Affiliations:** ^1^Department of Neurology, Mersin University, Mersin, Türkiye; ^2^Global Migraine and Pain Society, Istanbul, Türkiye; ^3^Bambino Gesù Children's Hospital (Istituti di Ricovero e Cura a Carattere Scientifico), Rome, Italy; ^4^Developmental Neurology Unit, Rome, Italy; ^5^Brain 360 Neurology Center, Istanbul, Türkiye; ^6^Unit of Child and Adolescent Neuropsychiatry, Sant'Andrea University Hospital, Sapienza, Rome, Italy

**Keywords:** cognitive schemas, frontostriatal dysfunction, migraine, cognitive behavioral therapy (CBT), migraine management

Headache disorders are prevalent among individuals of all age groups and have a significant impact on their overall quality of life and functioning. The cognitive schemas approach to understanding these disorders suggests that specific patterns of thinking, attitudes, and beliefs can perpetuate or worsen primary headaches. Recent studies have also identified a link between cognitive schema dysfunction and the development and persistence of primary headache disorders in adults. This approach is especially relevant in children and adolescents, where cognitive development and psychological factors may contribute to headache pathology (1).

Furthermore, recent research has revealed the role of frontostriatal dysfunction in the development and maintenance of headache disorders, including their association with common psychiatric comorbidities (2, 3). The frontostriatal network plays a critical role in regulating pain perception and emotional processing, and dysfunction within this network may contribute to the onset and continuation of primary headaches in young individuals (2, 4, 5).

Moreover, individuals with chronic headache disorders, such as migraines and tension-type headaches, have reported disturbances in their body schema (6). Schemas, which are the fundamental structures of cognition, have not received sufficient attention. Body schema refers to an individual's perception and awareness of their own body, and disruptions in body schema may contribute to chronic pain conditions, including headache disorders. Previous studies have examined early maladaptive schemas (EMSs) and the clinical characteristics of migraines in adolescents. Female adolescent migraineurs demonstrated significantly elevated scores for EMSs related to emotional deprivation, abandonment/instability, defectiveness/shame (disconnection/rejection domain), dependence/incompetence, vulnerability to harm/illness, failure (in impaired autonomy/performance domain), and negativity/pessimism (in hypervigilance/inhibition domain). Conversely, male migraineurs had significantly elevated scores only in insufficient self-control/self-discipline (in impaired limits domain). The type of migraine and current psychopathology did not significantly affect EMS domains, while a history of sexual abuse significantly influenced certain EMSs. Consequently, body schema therapy has been proposed as a potential treatment option, particularly for female migraine sufferers and those with chronic headache disorders (5, 6) (Güler Aksu et al.).

To effectively manage primary headache disorders, a multidisciplinary approach is recommended, which encompasses lifestyle adjustments, pharmacological interventions, and behavioral interventions such as cognitive-behavioral therapy (CBT) and body schema therapy. CBT has proven to be an effective behavioral intervention for managing primary headache disorders in adults (4), while body schema therapy has shown promising outcomes in improving pain perception, disability, and quality of life among individuals with chronic pain conditions (7, 8). CBT has also demonstrated efficacy as a behavioral intervention for managing primary headache disorders in children and adolescents (9). These interventions typically involve identifying and challenging negative cognitive schemas and maladaptive coping strategies, enhancing emotion regulation and stress management skills, and implementing relaxation techniques (8, 9).

Additionally, recent studies have underscored the potential of neuromodulation techniques, such as transcranial magnetic stimulation (TMS), in the management of primary headache disorders. TMS can modulate the activity of the frontostriatal network, thereby reducing pain severity (10).

This Research Topic seeks to provide a comprehensive understanding of cognitive schemas, frontostriatal dysfunction, and their potential roles in the development and persistence of primary headache disorders among children and adolescents. The contributions within this volume explore the application of CBT, neuromodulation techniques, and other behavioral interventions in managing primary headache disorders. Furthermore, they emphasize the significance of adopting a personalized approach to the management of primary headache disorders in adults. This approach involves identifying individual cognitive and body schema disturbances that contribute to headache pathology and tailoring treatment interventions accordingly. Moreover, recent studies have highlighted the importance of patient education and self-management strategies for the long-term management of primary headache disorders in adults (4, 10).

We trust that this Editorial provides readers with a comprehensive overview of the latest research on cognitive schemas, frontostriatal dysfunction, and primary headache disorders, as well as insights into the potential of multidisciplinary management approaches. We extend our sincere gratitude to the contributing authors for their valuable insights and efforts in compiling this collection of articles.

## Author contributions

AÖ submitted the paper. All authors contributed to the article and approved the submitted version.
